# A recurring packing contact in crystals of InlB pinpoints functional binding sites in the internalin domain and the B repeat

**DOI:** 10.1107/S2059798322000432

**Published:** 2022-02-18

**Authors:** Christina Geerds, Willem M. Bleymüller, Timo Meyer, Christiane Widmann, Hartmut H. Niemann

**Affiliations:** a Department of Chemistry, Bielefeld University, Universitätsstrasse 25, 33615 Bielefeld, Germany

**Keywords:** InlB, *Listeria monocytogenes*, binding sites, crystal contacts, crystallization propensity, protein–protein interactions

## Abstract

InlB, a bacterial agonist of the human receptor tyrosine kinase MET, consists of an N-terminal internalin domain, a central B repeat and three C-terminal GW domains. In all previous structures of full-length InlB or an InlB construct lacking the GW domains (InlB_392_), there was no interpretable electron density for the B repeat. Here, three InlB_392_ crystal structures in which the B repeat is resolved are described. These are the first structures to reveal the relative orientation of the internalin domain and the B repeat. A wild-type structure and two structures of the T332E variant together contain five crystallographically independent molecules. Surprisingly, the threonine-to-glutamate substitution in the B repeat substantially improved the crystallization propensity and crystal quality of the T332E variant. The internalin domain and B repeat are quite rigid internally, but are flexibly linked to each other. The new structures show that inter-domain flexibility is the most likely cause of the missing electron density for the B repeat in previous InlB structures. A potential binding groove between B-repeat strand β2 and an adjacent loop forms an important crystal contact in all five crystallographically independent chains. This region may represent a hydrophobic ‘sticky patch’ that supports protein–protein interactions. This assumption agrees with the previous finding that all known inactivating point mutations in the B repeat lie within strand β2. The groove formed by strand β2 and the adjacent loop may thus represent a functionally important protein–protein interaction site in the B repeat.

## Abstract 2

InlB, an invasion protein from the facultative intracellular pathogen *Listeria monocytogenes*, is a multi-domain protein that binds and activates the human receptor tyrosine kinase MET. Three new InlB crystal structures reveal the relative orientation of the first two domains (the internalin domain and the B repeat) for the first time. A recurrent packing contact formed by all five crystallographically independent molecules highlights functionally important binding sites in the internalin domain and the B repeat.

## 1. Introduction

InlB is an invasion protein that is located on the surface of the bacterium *Listeria monocytogenes* (Dramsi *et al.*, 1995 ▸; Lingnau *et al.*, 1995 ▸). As a facultative intracellular pathogen, *L. monocytogenes* can stimulate its own uptake by non­phagocytic cells such as epithelial or endothelial cells (Pizarro-Cerdá *et al.*, 2012 ▸). Binding of InlB to the receptor tyrosine kinase MET on host cells is one way to induce internalization of bacteria (Shen *et al.*, 2000 ▸). MET acts as a receptor for hepatocyte growth factor/scatter factor (HGF/SF) and is essential during mammalian development (Birchmeier *et al.*, 2003 ▸). MET also contributes to tissue regeneration and wound healing, while MET deregulation can promote cancer meta­stasis (Trusolino *et al.*, 2010 ▸). Cellular phenotypes in response to InlB stimulation resemble those observed upon MET activation by HGF/SF. They include cell motility (for example cell scatter of clonally growing MDCK or HT-29 cells) and cell proliferation (Shen *et al.*, 2000 ▸; Niemann *et al.*, 2007 ▸). At the molecular level, InlB induces MET phosphorylation and the activation of downstream signalling pathways such as phosphorylation of extracellular signal-regulated kinase (ERK) or activation of phosphoinositide 3-kinase (PI3K) and phosphorylation of protein kinase B (PKB)/AKT (Copp *et al.*, 2003 ▸; Seveau *et al.*, 2007 ▸).

**Figure 1:**
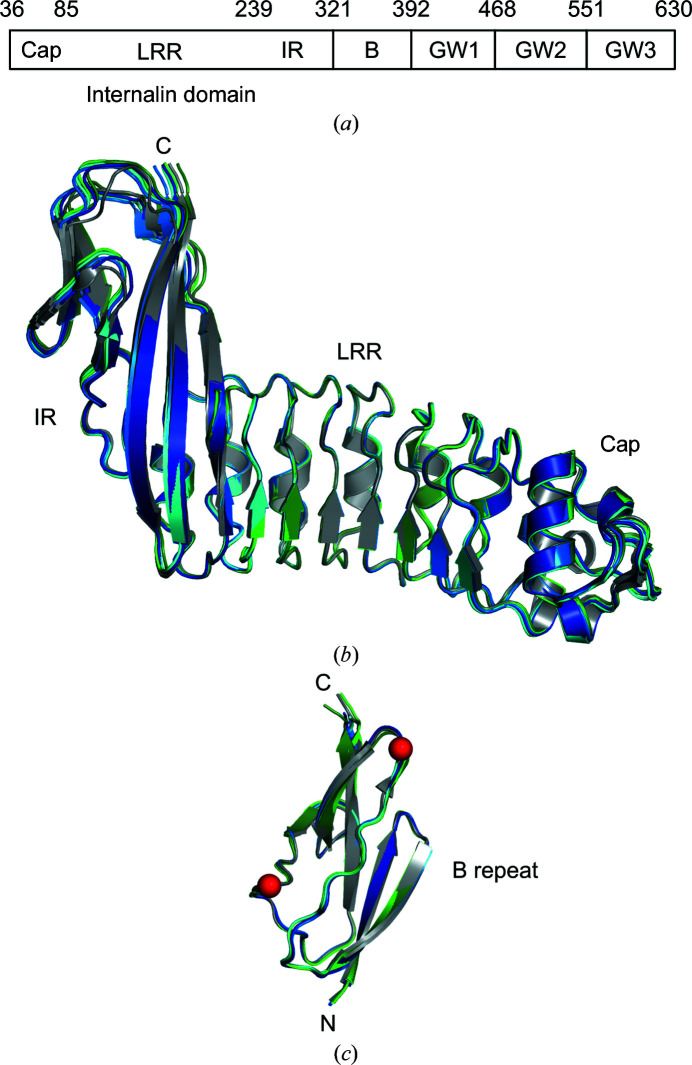
Domain structure and overlay of individual domains with previous high-resolution structures. (*a*) Domain structure of InlB. The amino-acid numbers shown above indicate domain boundaries. The internalin domain consists of three structural regions: cap, LRR and IR. (*b*, *c*) Chains *A*, *B* and *C* of InlB_392__wt are shown in dark blue, blue and cyan, respectively. Crystal forms I and II of InlB_392__T332E are shown in green and dark green, respectively. Reference structures are shown in grey. (*b*) The internalin domains of all InlB_392_ structures were overlaid on PDB entry 1h6t. The overlay was performed for the LRR region. (*c*) The B repeat of all InlB_392_ structures was overlaid on chain *A* of PDB entry 2y5p. The red spheres indicate the C^α^ atoms of the residues before (residue 353) and after (residue 373) the region that is not resolved in chain *C* of InlB_392__wt.

Mature InlB is a 595-amino-acid, multi-domain protein (Fig. 1 ▸ *a*). The N-terminal internalin domain (amino acids 36–321; InlB_321_) is characterized by a central leucine-rich repeat (LRR) region that binds to the first Ig-like IPT domain of MET with high affinity (Niemann *et al.*, 2007 ▸). The LRR region is flanked N-terminally by a helical cap and C-terminally by an Ig-like inter-repeat (IR) region (Schubert *et al.*, 2001 ▸). The C-terminal part (amino acids 393–630) consists of three GW domains named after a conserved Gly–Trp motif. The GW domains have an SH3-like fold and a high isoelectric point (pI) of about 10 (Marino *et al.*, 2002 ▸). The GW domains bind polyanions, including lipoteichoic acid (LTA), on the bacterial surface (Jonquières *et al.*, 1999 ▸) and heparan sulfate of host cell surface heparan sulfate proteoglycans (HSPGs; Jonquières *et al.*, 2001 ▸). Binding to LTA or HSPGs is mutually exclusive (Jonquières *et al.*, 2001 ▸). InlB apparently acts as a soluble protein that induces bacterial uptake into host cells rather than as an adhesin that supports the attachment of *L. mono­cytogenes* to host cells (Jonquières *et al.*, 2001 ▸; Banerjee *et al.*, 2004 ▸). Binding of the GW domains to host HSPGs enhances MET activation through the internalin domain (Banerjee *et al.*, 2004 ▸), presumably via an avidity effect or receptor clustering (Jonquières *et al.*, 2001 ▸; Niemann *et al.*, 2007 ▸). On their own or provided in *trans* with InlB_392_, the GW domains showed no effect in cellular assays (Banerjee *et al.*, 2004 ▸).

InlB activates MET by dimerization of the MET ecto­domain (Niemann, 2013 ▸). InlB-induced MET dimerization on cells was shown by fluorescence microscopy (Dietz *et al.*, 2013 ▸; Harwardt *et al.*, 2017 ▸; Koschut *et al.*, 2016 ▸). The crystal structure of a 2:2 complex formed by the InlB internalin domain and a large portion of the MET extracellular domain revealed an arrangement in which two InlB molecules form a twofold-symmetric homodimer via the convex distal surface of their LRR regions (Niemann *et al.*, 2007 ▸; Ferraris *et al.*, 2010 ▸). Two MET molecules sit on the outside of this InlB dimer, with each being bound to the concave ‘front side’ of the LRR region. Except for a small contact of two IPT2 domains, MET does not contribute to dimerization in this structure. The same 2:2 arrangement of the InlB internalin domain and MET has recently been observed in another structure obtained in the presence of a MET-binding DARPin, lending further support to this activation model (Andres *et al.*, 2019 ▸).

InlB structures have been published, in the following order, of the LRR fragment (InlB_248_; Marino *et al.*, 1999 ▸), the internalin domain (InlB_321_; Schubert *et al.*, 2001 ▸) and the full-length protein (Marino *et al.*, 2002 ▸). However, the B repeat was not resolved in the structure of full-length InlB. There was some electron density between the internalin and GW domains, but this could not be modelled. Therefore, we determined the structure of a fragment consisting of the internalin domain and the B repeat but lacking the GW domains (InlB_392_; Ebbes *et al.*, 2011 ▸). Again, the B repeat was not resolved and this time there was no electron density at all for the B repeat, suggesting it to be highly flexible. In this crystal form, all packing contacts are formed by the internalin domain. We assume that the B repeat makes no crystal contacts at all and that it is free to move in large solvent channels. Therefore, we crystallized the B repeat alone (Ebbes *et al.*, 2011 ▸). These crystals diffracted to high resolution (1.3 Å) and revealed a well defined structure. The B repeat folds into a compact domain resembling a β-grasp fold. The only difference from a canonical β-grasp fold is the absence of the helix connecting strands β2 and β3. Instead, the two parallel edge β-strands are connected by an extended loop structure. While structures of all domains of InlB are now available, the relative orientation of the B repeat and the internalin domain remains elusive.

The B repeat is arguably the least understood domain in InlB. Several internalins contain up to four domains that are homologous to the B repeat, yet none of them has been functionally characterized (Bierne *et al.*, 2007 ▸). Initial experiments with recombinant domain-deletion constructs of InlB showed that the B repeat contributes to ERK activation, but found no effect on MET phosphorylation (Copp *et al.*, 2003 ▸). This led to the suggestion that the B repeat could bind a co-receptor and activate a second signalling pathway parallel to MET. A more detailed study revealed that InlB_392_ has a slightly (about twofold) higher potency in inducing MET phosphorylation than InlB_321_ (Banerjee *et al.*, 2004 ▸). Thus, the B repeat apparently does contribute to MET activation. Cell-motility assays with canine MDCK and human cells revealed a profound effect of the B repeat. While InlB_392_ induced MDCK colony scatter at 10 n*M* (Ebbes *et al.*, 2011 ▸), InlB_321_ was unable to do so even at 1000 n*M* (Ferraris *et al.*, 2010 ▸). A construct consisting only of the B repeat and GW1–3 had no effect (Ebbes *et al.*, 2011 ▸). In human HT-29 cells, InlB_392_ was about tenfold more active than InlB_321_ (Bleymüller *et al.*, 2016 ▸). In summary, the B repeat on its own shows no effect on cells. When fused to the internalin domain it enhances MET phosphorylation. Its presence increases potency even more in assays of ERK or AKT phosphorylation or cellular phenotypes such as cell motility. So far, however, all of our efforts to identify the postulated co-receptor for the B repeat have failed (Bleymüller *et al.*, 2016 ▸).

Analysis of InlB_392_ variants with single or multiple substitutions on the surface of the B repeat in cellular receptor-activation assays revealed only two functionally important amino acids, both of which are located in the edge strand β2 (Bleymüller *et al.*, 2016 ▸). The mutation of Thr332 or Ile334 strongly impaired or completely abolished the activity of InlB_392_ in cellular assays. The resulting protein variants were at least 100-fold less active than wild-type InlB_392_ (InlB_392__wt). Interestingly, the InlB_392_ variants T332E and I334D/T336L (variant D in Bleymüller *et al.*, 2016 ▸) were inactive in a cell-motility assay with human HT-29 cells even at the highest concentration tested (1000 n*M*), while the internalin domain alone stimulated cell scatter at 10 n*M*. Thus, InlB_392_ variants with a single substitution in the B repeat are substantially less active than a construct that completely lacks the B repeat. One possible mechanistic explanation for this perplexing result is that the wild-type B repeat contributes to MET dimerization, whereas the T332E or I334D mutation hinders dimerization of the internalin domains (Bleymüller *et al.*, 2016 ▸). To scrutinize this hypothesis, it would be highly interesting to resolve the relative orientation of the internalin domain and to identify protein–protein interaction sites in the B repeat.

Here, we describe the crystallization and structure determination of wild-type InlB_392_ (InlB_392__wt) and the inactive variant InlB_392__T332E. While the wild-type protein was difficult to crystallize and the rare crystals generally showed no or rather low-resolution diffraction, we obtained crystals of InlB_392__T332E under several conditions and were able to determine two structures at resolutions of 2.05 and 1.8 Å. Both domains are resolved in all three structures, revealing the arrangement of the internalin domain and the B repeat in InlB for the first time.

## 2. Materials and methods

### 2.1. Protein crystallization

All three proteins were expressed and purified as described in Bleymüller *et al.* (2016 ▸). For crystallization, the protein buffer phosphate-buffered saline (PBS), in which the proteins were stored at −80°C, was exchanged for crystallization buffer (10 m*M* Tris pH 8.0, 20 m*M* NaCl). All proteins were crystallized in MRC 2-well plates with drops consisting of 100 nl protein (10 mg ml^−1^) and 100 nl reservoir solution set up using a Crystal Gryphon robot (Art Robbins Instruments). All crystals were harvested directly from commercial or homemade standard screens without further optimization. InlB_392__wt crystallized at 20°C in the MORPHEUS screen (Gorrec, 2009 ▸) condition E6 [0.1 *M* (HEPES sodium salt/MOPS acid) pH 7.5, 20%(*v*/*v*) ethylene glycol, 10%(*w*/*v*) PEG 8000, 30 m*M* diethylene glycol, 30 m*M* triethylene glycol, 30 m*M* tetraethylene glycol, 30 m*M* pentaethylene glycol]. For harvesting, crystals were cryoprotected in reservoir solution to which an additional 20%(*v*/*v*) ethylene glycol was added before flash-cooling the crystal in liquid nitrogen. Crystal form I of InlB_392__T332E was obtained at 4°C in the MORPHEUS screen condition G2 [0.1 *M* (imidazole/MES) pH 6.5, 20%(*v*/*v*) ethylene glycol, 10%(*w*/*v*) PEG 8000, 20 m*M* sodium formate, 20 m*M* ammonium acetate, 20 m*M* sodium citrate tribasic dehydrate, 20 m*M* sodium potassium tartrate tetrahydrate, 20 m*M* sodium oxamate]. These crystals were harvested directly from the crystallization drop and flash-cooled in liquid nitrogen without the addition of a cryoprotectant. Crystal form II of InlB_392__T332E was obtained at 4°C in condition D3 of a homemade PEG smear screen with low- and broad-molecular-weight PEGs as described in Chaikuad *et al.* (2015 ▸). The reservoir consisted of 0.1 *M* succinate pH 7.0, 0.2 *M* Li_2_SO_4_ and 22.5% PEG mixture consisting of equal amounts of eight low-molecular-weight PEGs (PEG 300, PEG 400, PEG 500 MME, PEG 550 MME, PEG 600, PEG 750 MME, PEG 1000 and PEG 1000 MME). These crystals were cryoprotected in reservoir solution additionally containing 15% glycerol before flash-cooling in liquid nitrogen.

### 2.2. Data collection and processing

Data for InlB_392__wt were collected on beamline P13 operated by EMBL Hamburg at the PETRA III storage ring (Cianci *et al.*, 2017 ▸) using a PILATUS 6M detector (Dectris). For InlB_392__T332E, measurements were carried out on the BL14.2 beamline at the BESSY II electron-storage ring operated by the Helmholtz-Zentrum Berlin für Materialien und Energie (Mueller *et al.*, 2015 ▸). Data for both crystal forms of InlB_392__T332E were collected on a PILATUS 3S 2M detector (Dectris). Between 240 and 360° of fine-sliced data (0.1° per frame) were collected. The data were indexed and integrated with *XDS* (Kabsch, 2010 ▸) and scaled with *XSCALE* using zero-dose extrapolation (Diederichs *et al.*, 2003 ▸). Data-collection statistics are shown in Table 1 ▸.

### 2.3. Structure determination and refinement

The structures were solved by molecular replacement with *Phaser* (McCoy *et al.*, 2007 ▸) from the *CCP*4 package (Winn *et al.*, 2011 ▸). The internalin domain (PDB entry 1h6t; Schubert *et al.*, 2001 ▸) was placed first, followed by placement of the B repeat (PBD entry 2y5p; Ebbes *et al.*, 2011 ▸). The structures were completed by iterative cycles of model building in *Coot* (Casañal *et al.*, 2020 ▸) and refinement in *REFMAC*5 (Kovalevskiy *et al.*, 2018 ▸) during the early stages and *phenix.refine* (Liebschner *et al.*, 2019 ▸) during the final stages of rebuilding. TLS refinement was used for all three structures. For InlB_392__wt with three chains in the asymmetric unit, local non­crystallographic symmetry (NCS) restraints and restraints to InlB_392__T332E crystal form II (PDB entry 7nms) as a reference model were applied. Refinement statistics are shown in Table 1 ▸.

## 3. Results

### 3.1. Crystallization and structure determination

We extensively screened for new crystallization conditions of wild-type InlB_392_. Crystals only grew in condition E6 of a MORPHEUS screen (Gorrec, 2009 ▸) stored beyond its ‘use by’ date. We were unable to reproduce or optimize these crystals with homemade solutions. At 20°C we obtained single crystals shaped as hexagonal plates typically of about 60 × 60 × 10 µm in size and reaching up to 100 × 100 × 15 µm. These crystals showed no diffraction even on beamlines BL14.2 of BESSY II and P13 of PETRA III. At 4°C we obtained thin rod-shaped crystals with a cross-section of about 10 × 10 µm and a length of 80–100 µm. The best crystal diffracted to 3.3 Å resolution (Table 1 ▸). For flash-cooling, we had to add ethylene glycol (see Section 2) as the reservoir solution alone did not freeze clearly, although all conditions of the MORPHEUS screen should be inherently cryoprotected.

InlB_392__T332E, an InlB_392_ variant with a single Thr-to-Glu substitution in the B repeat, readily yielded single crystals of varying morphologies in several conditions from the commercial MORPHEUS screen and a homemade PEG smear screen (Chaikuad *et al.*, 2015 ▸). These crystals reached a size and quality sufficient for data collection without further optimization. The best crystal from condition G2 of the MORPHEUS screen (crystal form I) was a rhombic plate with a cross-section of about 125 × 50 µm and diffracted to 2.05 Å resolution (Table 1 ▸). The best crystal from condition D3 of the PEG smear (low- and broad-molecular-weight) screen (crystal form II) was a rod with a cross section of about 35 × 35 µm and a length of about 180 µm. This crystal diffracted to 1.8 Å resolution (Table 1 ▸). We also collected data from InlB_392__T332E crystals grown under different conditions from the MORPHEUS or PEG smear screens. Some were isomorphous to crystal form I and diffracted to lower resolution, so we did not pursue structure determination. Others had larger unit cells and showed signs of translational non­crystallographic symmetry in the native Patterson map. Our attempts to solve these structures by molecular replacement in *Phaser* both with and without the tNCS option have so far failed.

All three structures described in this paper were easily solved by molecular replacement. *Phaser* was able to place both the internalin domain and the B repeat for all five crystallo­graphically independent molecules. The refinement statistics are listed in Table 1 ▸. The presence of many weak reflections due to translational pseudosymmetry (see below) may explain the relatively high *R*_work_ and *R*_free_ values for InlB_392__wt.

### 3.2. Overall structure

As expected, there are no major differences between the internalin domains in the InlB_392_ structures and the previously reported structures of the InlB internalin domain. An overlay of the LRR region revealed some flexibility in the cap and especially in the IR region (Fig. 1 ▸ *b*). Likewise, there are no major differences between the B repeats in the InlB_392_ structures and the published high-resolution structure of the isolated B repeat, with the exception of chain *C* in the InlB_392__wt structure, which shows no clear density for residues 354–372 corresponding to strand β3 and the following loop in the other B-repeat structures (Fig. 1 ▸ *c*). These residues may be flexible or adopt multiple conformations which cannot be resolved at this rather low resolution of 3.3 Å.

**Figure 2:**
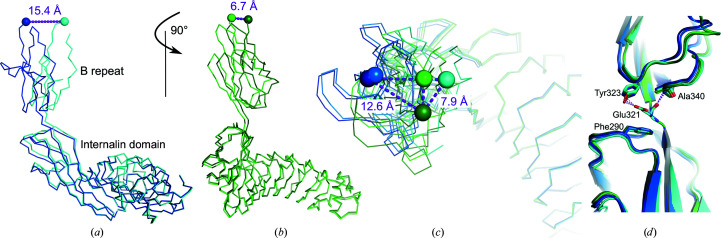
Movement of the B repeat relative to the internalin domain. Colouring is as in Fig. 1 ▸. Chains *A*, *B* and *C* of InlB_392__wt are shown in dark blue, blue and cyan, respectively. Crystal forms I and II of InlB_392__T332E are shown in green and dark green, respectively. The C^α^ atoms of the C-terminal residues 391 are shown as spheres. Dashed purple lines indicate the distances between C-terminal residues. (*a*) Chains *A* (dark blue) and *C* (cyan) of InlB_392__wt were aligned on the LRR region. (*b*) The view is rotated relative to (*a*) by 90° around a vertical axis. Crystal forms I (green) and II (dark green) of InlB_392__T332E were aligned on the LRR region. (*c*) Overlay of all InlB_392_ structures aligned on the LRR region and shown from the top of the B repeat. (*d*) Residue Glu321 appears to be an integral part of the B repeat (top) rather than the internalin domain (bottom). Glu321 forms hydrogen bonds from its side chain to the hydroxyl group of Tyr323 and from its backbone carbonyl to the backbone N atom of Ala340. The only interaction with the internalin domain is a CH–π interaction with Phe290.

The orientation of the B repeat relative to the internalin domain varies substantially (Figs. 2 ▸ *a*–2 ▸ *c*). There are no polar contacts between the internalin domain and the B repeat to stabilize their arrangement. Many internalins have at least one domain C-terminal to the LRR or IR region (Bierne *et al.*, 2007 ▸). Besides InlB, InlK is the only internalin for which a structure extending beyond the internalin domain is known (Neves *et al.*, 2013 ▸). The LRR-adjacent D2 domain and the following D3 domain of InlK are structurally distinct from the InlB IR region and B repeat, respectively. Nevertheless, as in our InlB_392_ structures, few contacts and large flexibility between D2 and D3 were found in InlK (Neves *et al.*, 2013 ▸). In InlB, the short linker between the internalin domain and the B repeat acts as a pivot point rather than a hinge (Figs. 2 ▸ *a*–2 ▸ *c*).

Glu321 was previously regarded as part of the internalin domain (Schubert *et al.*, 2001 ▸), but may rather belong to the B repeat, as it forms two polar contacts with other B-repeat residues but none with the internalin domain. In all five crystallographically independent InlB_392_ chains reported here there are hydrogen bonds between the backbone carbonyl O atom of Glu321 and the backbone NH of Ala340 and between the side-chain carboxylate of Glu321 and the phenolic OH of Tyr323 (Fig. 2 ▸ *d*). The only interaction of Glu321 and the internalin domain is a CH–π interaction of the aliphatic part of the glutamate side chain with the aromatic ring of Phe290.

### 3.3. Crystal packing

**Figure 3:**
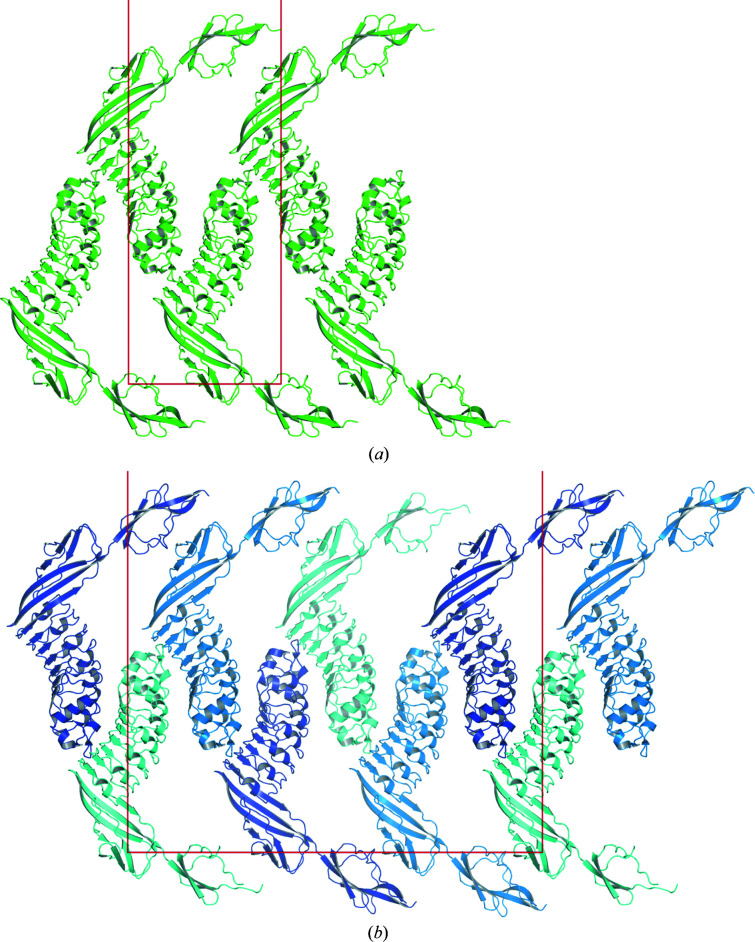
Crystal packing. (*a*) View along the *a* axis of InlB_392__T332E crystal form I. The *b* axis and part of the *c* axis (54.59 and 226.72 Å) are shown horizontally and vertically, respectively. There is one molecule per asymmetric unit. (*b*) View along the *a* axis of InlB_392__wt. The *b* axis and part of the *c* axis (148.41 and 220.02 Å) are shown horizontally and vertically, respectively. There are three molecules per asymmetric unit related by translational noncrystallographic symmetry. The translational component along *b* is close to 1/3. Chains *A*, *B* and *C* are shown in dark blue, blue and cyan, respectively.

None of the structures contained a twofold-symmetric arrangement of InlB_392_ indicative of a dimer, and the *PISA* server (Krissinel & Henrick, 2007 ▸) predicted InlB_392_ to be monomeric in solution for all three structures. The packing of InlB_392__T332E crystal form I is closely related to that of InlB_392__wt and the surroundings of all three molecules in the wild-type structure are similar. In a way, the packing of InlB_392__wt can be viewed as a slightly distorted version of InlB_392__T332E crystal form I with a tripled *b* axis (Fig. 3 ▸). Accordingly, the native Patterson map of InlB_392__wt has an off-origin peak with a peak height of 41.7% of the origin peak at coordinates *u*, *v*, *w* = 0.0000, 0.3234, 0.0000 according to the tNCS detection of *Phaser* (38.0% at 0.000, 0.322, 0.000 according to *phenix.xtriage*). The packing of InlB_392__T332E crystal form II is different.

A common contact of all four molecules of InlB_392__wt and InlB_392__T332E crystal form I is formed between the concave LRR side of one molecule and the cap region and the convex LRR side of another molecule (Fig. 3 ▸). The concave side of the LRR is the primary binding site for the MET receptor (Niemann *et al.*, 2007 ▸). The crystal contact involves exposed aromatic side chains of InlB that are essential for MET binding (Machner *et al.*, 2003 ▸). The contact area is between 397 and 647 Å^2^, with a mean of 549 Å^2^, according to the *PISA* server (Krissinel & Henrick, 2007 ▸). This crystal contact is not present in crystal form II of InlB_392__T332E.

**Figure 4:**
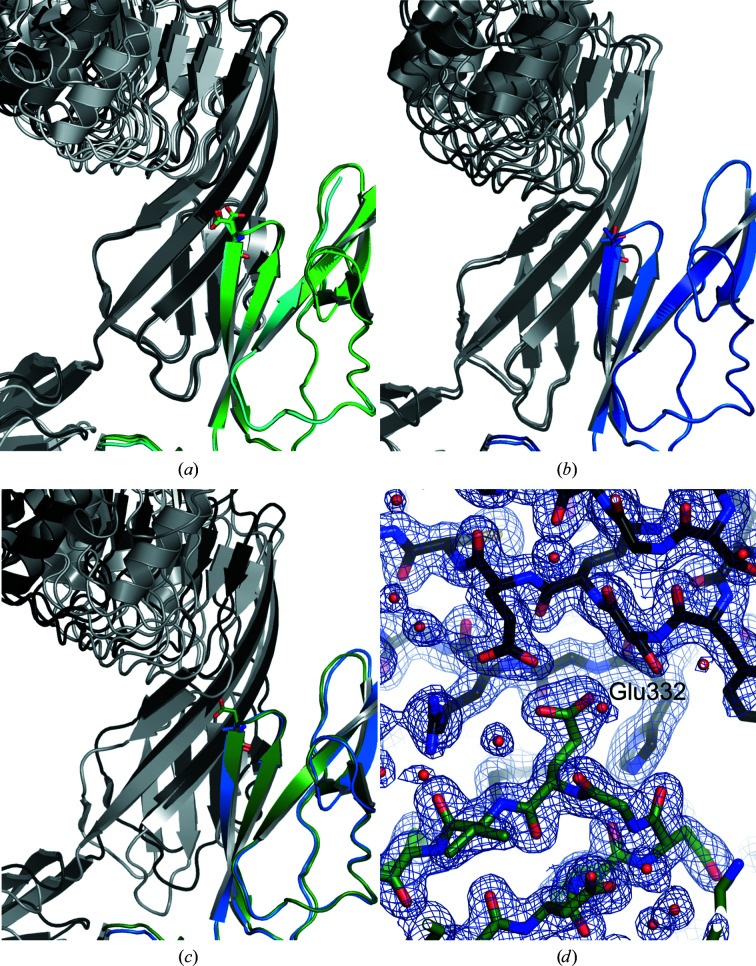
All crystallographically independent InlB_392_ molecules form a crystal contact between their B repeat and a symmetry-related IR region. B repeats are structurally aligned and colour-coded as in Fig. 1 ▸. Symmetry-related molecules are shown in grey. (*a*) The same contact is formed in both crystal forms of InlB_392__T332E and in InlB_392__wt between the B repeat of chain *C* and the IR region of chain *A*. The B repeat and the symmetry-related InlB_392_ are coloured as follows: InlB_392__T332E crystal form I (PDB entry 7pv8), green and medium grey; InlB_392__T332E crystal form II (PDB entry 7nms), dark green and dark grey; InlB_392__wt (PDB entry 7pv9; chains *C* and *A*), cyan and light grey. (*b*) A similar contact is formed in InlB_392__wt between the B repeat of chain *A* or *B* and the IR region of chain *C* or *B*, respectively. The B repeat and the symmetry-related InlB_392_ are coloured as follows: chains *B*, blue and medium grey; chains *A* and *C*, dark blue and dark grey. (*c*) An overlay of the B repeats of InlB_392__wt chain *A* (blue) and InlB_392__T332E crystal form II (dark green) shows that the IR regions of the symmetry-related molecules are shifted. (*d*) Contact of the B repeat (C atoms in dark green) and a symmetry-related IR region (carbons in dark grey) of InlB_392__T332E crystal form II. 2*mF*_o_ − *DF*_c_ electron density is contoured at 1σ.

However, all five crystallographically independent InlB_392_ molecules in the three crystal forms have one recurring crystal contact in common. The contact area lies between 594 and 687 Å^2^, with a mean of 621 Å^2^, according to the *PISA* server (Krissinel & Henrick, 2007 ▸). This contact is formed between the B repeat of one molecule and the IR region of a neighbouring molecule (Fig. 4 ▸). On the B repeat it involves residues from strand β2 (mainly Val329, Thr/Glu332, Val333 and Ile334) and the long loop (residues 347–353) connecting strands β2 and β3 that is a helix in canonical β-grasp fold proteins. The *PISA* server calculates a favourable interaction for this contact (negative solvation-energy effect Δ^i^ *G* ranging from −10.0 to −23.4 kJ mol^−1^; mean −15.1 kJ mol^−1^), while the previously described LRR–cap contact has a positive Δ^i^ *G* ranging from −0.4 to 16.7 kJ mol^−1^ (mean 5.4 kJ mol^−1^). The overall arrangement of the contact between the B repeat and the IR region is similar for all five instances of InlB_392_ described in this paper. The precise geometry varies somewhat and two groups can be distinguished. One group has three members, namely both crystal forms of InlB_392__T332E and the B repeat of chain *C* packing against the IR region of chain *A* in InlB_392__wt (Fig. 4 ▸ *a*). The second group comprises two crystal contacts of InlB_392__wt, namely the B repeat of chain *B* packing against the IR region of a symmetry-related chain *B* and the B repeat of chain *A* packing against the IR region of chain *C* (Fig. 4 ▸ *b*). The different geometry of the two contact groups is illustrated in Fig. 4 ▸(*c*). In the T332E variant, the mutated residue Glu332 substantially contributes to formation of this crystal contact and is well defined in the electron density (Fig. 4 ▸ *d*).

Taking into account the packing of all three crystal forms described in this paper, the binding of the IR region to the groove between B-repeat strand β2 and the loop connecting β2 and β3 is clearly the dominant packing inter­action. This crystal contact has the largest mean interface area, the most negative Δ^i^ *G* and is formed by all molecules. Potential functional implications will be considered in Section 4.

## 4. Discussion

### 4.1. The T332E mutation has no impact on the B-repeat structure

The T332E substitution is one of two point mutations that we had previously found to have a negative effect on the biological function of the B repeat (Bleymüller *et al.*, 2016 ▸). Thr332 is surface-exposed. Therefore, we had expected the mutation to glutamate not to impair the B-repeat structure. The circular-dichroism spectrum and the elution behaviour on a gel-filtration column confirmed this assumption, as they were basically identical for the wild-type B repeat and the T332E variant (Bleymüller *et al.*, 2016 ▸). The crystal structures presented in this work provide additional and conclusive proof that the T332E mutation does not change the structure of the B repeat. Therefore, the negative effect of this substitution in cellular assays is most likely due to the destruction of a binding site, preventing the interaction with a functionally important binding partner.

### 4.2. Effect of the T332E mutation on crystallization

Intriguingly, wild-type InlB_392_ was a much more problematic crystallization target than the InlB_392__T332E variant. This is unexpected because glutamate side chains are statistically underrepresented in interfaces of oligomeric proteins, presumably due to their high conformational entropy (Derewenda & Vekilov, 2006 ▸). Moreover, crystal contacts are systematically depleted of residues with high side-chain entropy, and Glu, along with Lys, has the lowest propensity to form crystal contacts (Cieślik & Derewenda, 2009 ▸). In the semi-rational surface-entropy reduction (SER) approach, surface-exposed glutamates are mutated to alanine, threonine or tyrosine in order to increase the likelihood of crystallization (Mateja *et al.*, 2002 ▸; Cooper *et al.*, 2007 ▸). Analysis of the actual crystal contacts with the *PISA* server indicates an unfavourable contribution of Thr332 (positive solvation-energy effect Δ^i^ *G* ≃ 4.2 kJ mol^−1^) but an almost neutral effect of Glu332 (Δ^i^ *G* ≃ 0 kJ mol^−1^). One explanation for this unexpected effect of the T332E substitution would be that the B repeat evolved to prevent fortuitous binding to the IR domain and that the T332E mutation counteracts this anti-aggregation property.

### 4.3. Comparison with the structure of full-length InlB

**Figure 5:**
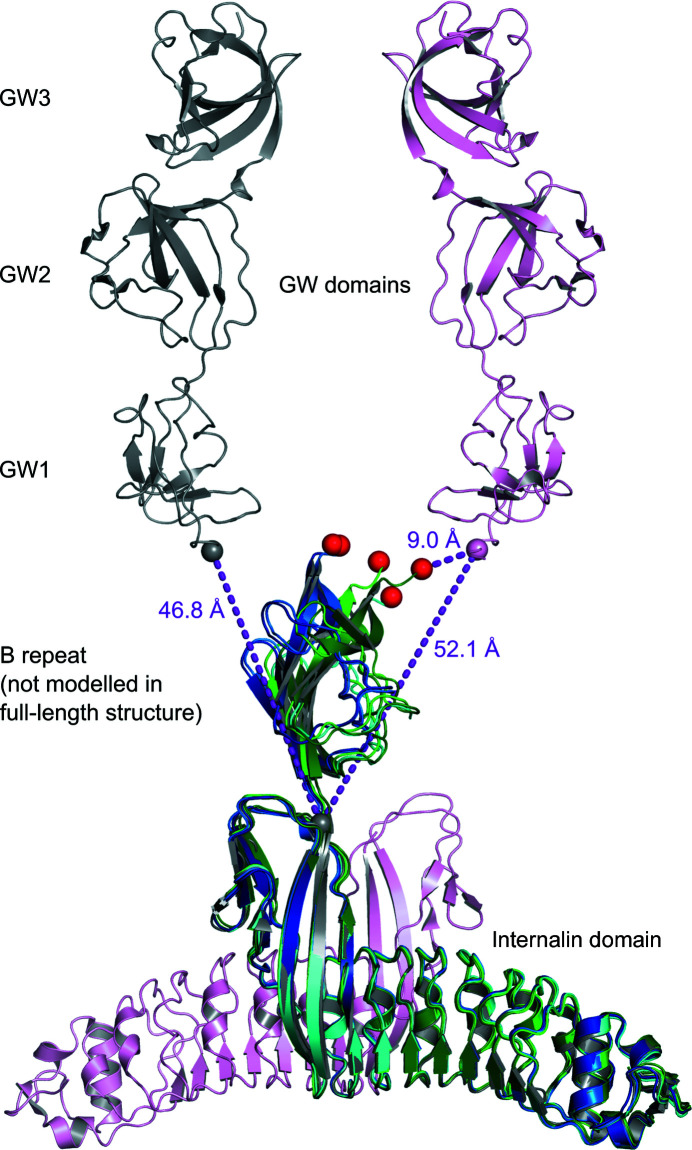
Comparison of InlB_392_ structures with the structure of full-length InlB (PDB entry 1m9s). Two symmetry-related copies of full-length InlB are shown in grey and pink, with the internalin domains at the bottom and the three GW domains at the top. C^α^ atoms of the last residue of the grey internalin domain (residue 319) and of residue 393 at the beginning of the GW domains are shown as spheres. Because there was no interpretable density for the B repeat, it was unclear which GW domains should be grouped with the grey internalin domain. Ghosh and coworkers deposited the asymmetric unit containing a combination of the internalin domain and GW domains with the shorter distance. The other choice of asymmetric unit (grey internalin domain and pink GW domains) would result in only a slightly longer distance. All InlB_392_ structures were structurally aligned on the grey internalin domain. Colouring is as in Fig. 1 ▸. Chains *A*, *B* and *C* of InlB_392__wt are shown in dark blue, blue and cyan, respectively. Crystal forms I and II of InlB_392__T332E are shown in green and dark green, respectively. C^α^ atoms of the C-terminal residues (391 or 392) are shown as red spheres. Crystal form II of InlB_392__T332E has the shortest distance of 9.0 Å between its C-terminal residue 392 and residue 393 of the pink GW domains. All distances are shown as purple dashed lines.

In the structure of full-length InlB there were two possibilities to connect the GW domains to the internalin domain, with similar distances between the C-terminal residue of the internalin domain and the N-terminal residue of the GW domains (Marino *et al.*, 2002 ▸). The authors deposited an asymmetric unit (shown in grey in Fig. 5 ▸) containing the combination with the slightly shorter distance of 47 Å between the C^α^ atoms of residues 319 and 393 in the Protein Data Bank. The distance to residue 393 of a symmetry-related copy of the GW domains (shown in pink in Fig. 5 ▸) is 52 Å.

Upon overlaying all five copies of InlB_392_ described in this work with one copy of the InlB full-length dimer, the C-terminal residues of the B repeat are about halfway between the two possible GW domains (Fig. 5 ▸). For chain *A* of InlB_392__wt, the distances to residue 393 of the GW domains of the deposited asymmetric unit and the symmetry-related GW domains are 22.1 and 20.8 Å, respectively. The largest difference between these distances is found for InlB_392__T332E crystal form II. The distances to residue 393 of the GW domains of the deposited asymmetric unit and the symmetry-related GW domains of PDB entry 1m9s (Marino *et al.*, 2002 ▸) are 34.4 and 9.0 Å, respectively. For all five instances of InlB_392_ reported here, the shortest distance between the C-terminal residue of the B repeat and the N-terminal residue of the GW domains is found for the symmetry-related GW domains. However, none of the orientations of the B repeat observed in InlB_392_ can represent the position present in the full-length protein in PDB entry 1m9s, as no residues are left that could span the distance of at least 9.0 Å. Moreover, if one overlays two copies of InlB_392_ onto the two copies of the functional full-length dimer, there would be severe clashes of the B repeat (Supplementary Fig. S1). Given the highly flexible linkage between the internalin domain and the B repeat, this suggests that crystal-packing forces govern the observed orientations of the B repeat.

### 4.4. Strand β2 of the B repeat may be a ‘sticky patch’ favouring cohesive interactions

**Figure 6:**
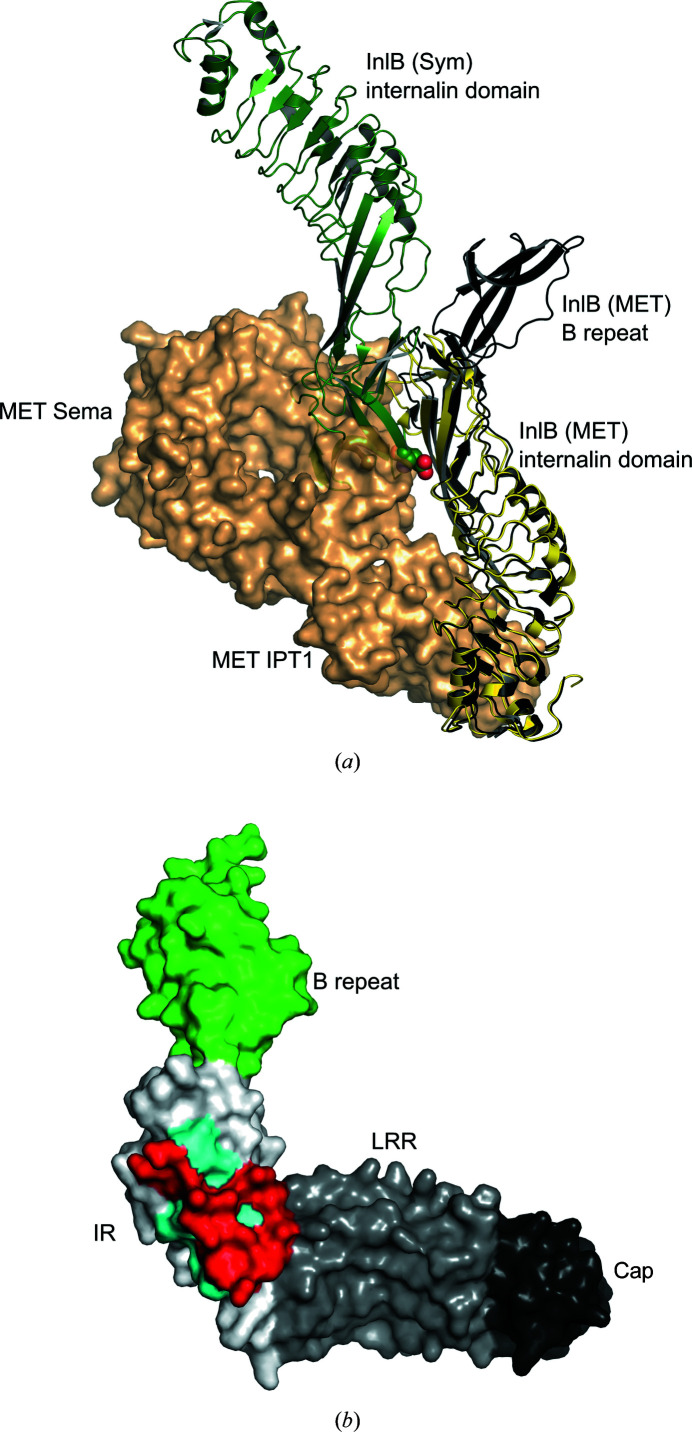
The same residues of the IR region bind the MET Sema domain in the InlB–MET complex and form the crystal contact to the B repeat in InlB_392_. (*a*) Overlay of the InlB–MET complex (PDB entry 2uzx) and InlB_392_ (PDB entry 7nms). MET is shown as an orange surface. InlB_392_ (grey) was structurally aligned with the InlB internalin domain (yellow) from the InlB–MET complex. A symmetry-related InlB_392_ is shown in dark green. The B repeat of this symmetry-related InlB_392_ overlaps with the MET Sema domain where it contacts the IR region. Glu332 that is mutated in the T332E variant is shown as spheres. (*b*) InlB_392_ (PDB entry 7nms) is shown as a surface. The cap region is coloured black, the LRR region grey, the IR region white and the B repeat green. Residues of the IR region involved in both binding of the MET Sema domain and formation of the crystal contact with the B repeat are shown in red. Residues only involved in the crystal contact with the B repeat are coloured cyan.

Strikingly, the contact between B-repeat strand β2 and the IR region of a neighbouring molecule is similar in all five chains. This is unusual as monomeric proteins rarely have common interfaces in more than one-third of their crystal forms and generally much less (Xu *et al.*, 2008 ▸). Recurring crystal contacts indicate energetically favourable interactions and sometimes they are even biologically relevant (Xu *et al.*, 2008 ▸). The contact between strand β2 and the IR region most likely does not represent a physiological contact. Firstly, in the complex with the MET receptor (Niemann *et al.*, 2007 ▸) the IR region contacts the Sema domain of the MET receptor with residues that contact strand β2 of the B repeat in the InlB_392_ structures (Fig. 6 ▸). Upon binding to MET, the IR region will therefore not be able to form this contact with the B repeat. Secondly, the contact between the B repeat and the IR region is formed regardless of the T332E mutation. As the T332E mutation strongly impairs the biological function of the B repeat in cellular assays, it appears highly unlikely that this represents a physiologically relevant contact.

**Figure 7:**
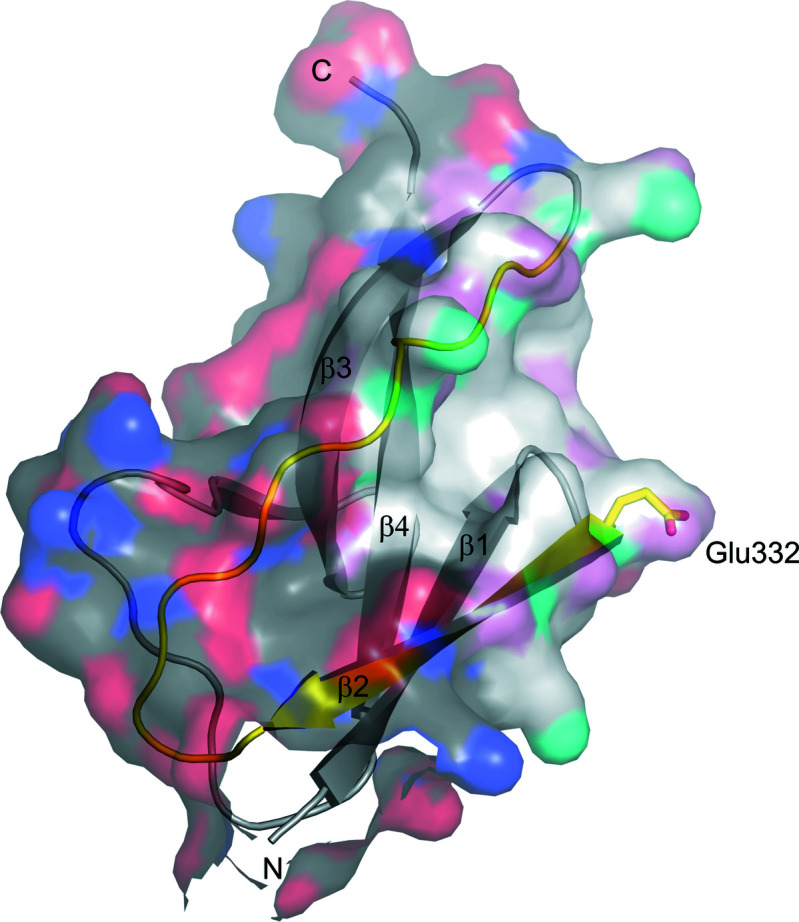
B-repeat residues forming the crystal contact to the IR region may belong to a ‘sticky patch’ in the hydrophobic groove between strand β2 and the following loop (both coloured yellow in the cartoon). The B repeat of InlB_392__T332E (PDB entry 7nms) is shown in cartoon representation with a transparent surface. The surface is coloured according to element, with C atoms in dark grey, N atoms in blue and O atoms in red. Residues involved in formation of the crystal contact with the IR region in InlB_392__T332E (PDB entry 7nms) are shown in lighter colours with C atoms in white, N atoms in cyan and O atoms in pink. The white patch highlights the apolar nature of the groove between strand β2 and the following loop.

Instead, we assume that this is a fortuitous interaction between two binding sites lacking their native binding partner. The MET binding site in the IR region and strand β2 of the B repeat may thus represent ‘sticky patches’, *i.e.* specific surface patches with properties that are thermodynamically favourable for cohesive interactions (Derewenda & Godzik, 2017 ▸). This hypothesis is supported by the Δ^i^ *G* *P*-value reported by the *PISA* server. The Δ^i^ *G* *P*-value is a measure of interface specificity. A *P*-value larger than 0.5 means that the interface is less hydrophobic than it could be and therefore the interface is likely to be an artefact of crystal packing. A *P*-value smaller than 0.5 indicates an interface with a hydrophobicity that is higher than the average for the given structures would be, implying that the interface surface can be interaction-specific (Krissinel & Henrick, 2007 ▸). The cap–LRR interface that is present in crystal form I of the T332E variant and three times in the wild-type structure has a mean Δ^i^ *G* *P*-value of 0.62, indicating a pure crystal-packing contact. In contrast, the interface between the B repeat and the IR region that is present in all structures has a Δ^i^ *G* *P*-value of 0.28, indicating higher hydrophobicity than would be expected on average (Fig. 7 ▸).

We previously observed a similar phenomenon for the *Y. enterocolitica* type III secretion protein SycD. Recombinantly produced SycD showed weak, concentration-dependent homodimerization in solution. Two crystal forms showed putative homodimers; however, the arrangement of protomers in these dimers differed (Büttner *et al.*, 2008 ▸). Both homodimers involved similar surface patches in the first tetratricopeptide repeat (TPR), but the geometry of the two contacts differed. Subsequently, a structure of the complex of the *Aeromonas hydrophila* homolog AscH with AopB suggested that SycD probably employs this region of TPR1 for interaction with its binding partner YopB, when present (Nguyen *et al.*, 2015 ▸). In the absence of YopB, the YopB binding site in TPR1 of SycD could be a ‘sticky patch’ that promotes fortuitous SycD homodimerization and supports different geometries of the dimer interface.

Our assumption that the hydrophobic groove formed by strand β2 and the loop connecting strands β2 and β3 forms the primary ligand-binding site in the B repeat (Fig. 7 ▸) is supported by our previous observations. In an attempt to identify potential binding sites in the B repeat by mutagenesis, the only two substitutions of surface residues that resulted in a loss of function were located in strand β2. We had suggested that the B repeat potentiates MET activation by forming a weak homodimer contact through which it could promote the dimerization of MET bound to the internalin domain. The T332E mutation would prevent homodimerization of the B repeat and thereby suppress MET dimerization through the internalin domain (Bleymüller *et al.*, 2016 ▸). The InlB_392_ structures presented here are compatible with the role of the B repeat in MET activation that we proposed previously. However, additional experiments will be required to corroborate or disprove this model, as we did not observe homodimerization of the B repeat even in crystals of wild-type InlB_392_.

## 5. Conclusion: crystal contacts as valuable assets

While crystal-packing contacts are often suspected to lead to structural artefacts, they can also help to reveal functional information when comparing different packing environments. Here, five crystallographically independent molecules reveal protein dynamics, highlighting the high inter-domain flexibility between the internalin domain and the B repeat of InlB. However, the five structures presented here apparently do not completely map out the conformational space of InlB_392_, as the B repeat needs to adopt yet another position to fit into the structure of full-length InlB. Therefore, additional crystal forms and further structures are likely to show even larger inter-domain movements.

The largest packing contact that is similarly formed by all five crystallographically independent instances of InlB_392_ involves the known binding site for the MET Sema domain in the IR region and a surface patch in the B repeat that has previously been shown to be functionally important through mutagenesis and cellular assays. The structures analysed in this paper thus represent a good example showing that recurrent crystal contacts can highlight physiologically relevant binding sites. Due to the extremely high protein concentration in crystals, crystallography allows the visualization of protein–protein interactions that are too weak to be studied in solution. The biological relevance of such contacts needs to be addressed by complementary functional assays (Kobe *et al.*, 2008 ▸). If an actual binding partner is missing, the interface area can still show up as a crystal contact interacting with heterologous protein surfaces in a nonphysiological way (Forwood *et al.*, 2007 ▸). In a time where protein crystallography is increasingly sandwiched between accurate protein structure prediction (Baek *et al.*, 2021 ▸; Jumper *et al.*, 2021 ▸) and high-resolution cryo-EM of large proteins and complexes (Kühlbrandt, 2014 ▸), the careful analysis of crystal contacts might turn out to be a valuable asset for crystallography.

## Supplementary Material

PDB reference: InlB_392_, wild type, 7pv9

PDB reference: InlB_392__T332E, crystal form I, 7pv8

PDB reference: crystal form II, 7nms

. DOI: 10.1107/S2059798322000432/jb5037sup1.pdf
crystal form II, 7nms

## Figures and Tables

**Table 1:** Data-collection and refinement statistics Values in parentheses are for the highest resolution shell.

		InlB_392__T332E	
	Wild-type InlB_392_	Crystal form I	Crystal form II
PDB code	7pv9	7pv8	7nms
Data collection			
Diffraction source	P13, PETRA III, DESY	BL14.2/MX2, BESSY	BL14.2/MX2, BESSY
Wavelength (Å)	0.9164	0.9184	0.9184
Detector	PILATUS 6M	PILATUS 3S 2M	PILATUS 3S 2M
Crystal-to-detector distance (mm)	422	253	223
Rotation range per image (°)	0.1	0.1	0.1
Total rotation range (°)	240	360	360
Space group	*P*2_1_2_1_2_1_	*P*2_1_2_1_2_1_	*P*2_1_2_1_2_1_
*a*, *b*, *c* (Å)	44.49, 148.41, 220.02	44.58, 54.59, 226.72	43.41, 88.73, 102.02
Mosaicity (°)	0.40	0.11	0.11
Resolution range (Å)	50.0–3.3 (3.39–3.30)	50.0–2.05 (2.10–2.05)	50.0–1.8 (1.85–1.80)
Total No. of reflections	195018 (14059)	470063 (34805)	492829 (36637)
No. of unique reflections	22938 (1677)	35861 (2598)	37383 (2714)
Completeness (%)	99.9 (99.9)	99.9 (100.0)	99.9 (100.0)
Multiplicity	8.5 (8.4)	13.5 (13.8)	13.2 (13.5)
〈*I*/σ(*I*)〉	6.49 (1.96)	16.56 (1.74)	23.59 (1.88)
*R*_meas_ (%)	34.0 (120.5)	12.2 (195.6)	6.9 (159.9)
CC_1/2_	0.992 (0.600)	0.999 (0.792)	1.000 (0.807)
Refinement			
Resolution range (Å)	48.27–3.30 (3.45–3.30)	16.15–2.05 (2.11–2.05)	26.77–1.80 (1.85–1.80)
No. of reflections, working set	21712 (2572)	33908 (2533)	35359 (2556)
No. of reflections, test set	1112 (126)	1754 (144)	1871 (127)
Final *R*_work_	0.2303 (0.3103)	0.1864 (0.2995)	0.1713 (0.2901)
Final *R*_free_	0.2723 (0.3579)	0.2287 (0.3683)	0.2175 (0.3541)
No. of non-H atoms	8279	3117	3218
R.m.s. deviations			
Bond lengths (Å)	0.003	0.008	0.004
Bond angles (°)	0.631	0.829	0.647
Average *B* factors (Å^2^)			
Protein	61	47	39
Other	n.a.	41	72
Water	n.a.	49	43
Ramachandran plot			
Most favoured (%)	97.02	96.62	96.90
Allowed (%)	2.98	3.38	3.10
Outliers (%)	0.00	0.00	0.00
