# Supplementary material for: A recurring packing contact in crystals of InlB pinpoints functional binding sites in the internalin domain and the B repeat
Source: Acta Crystallogr D Struct Biol. 2022 Feb 18;78(Pt 3):310–20. doi: 10.1107/S2059798322000432 (PMC8900821; doi:10.1107/S2059798322000432)
Supplement: Supplementary file 1 [file d-78-00310-sup1.pdf]

STRUCTURAL BIOLOGY

Volume 78 (2022)

Supporting information for article:

A recurring packing contact in crystals of InlB pinpoints functional binding sites in the internalin domain and the B repeat

Christina Geerds, Willem M. Bleymüller, Timo Meyer, Christiane Widmann and Hartmut H. Niemann

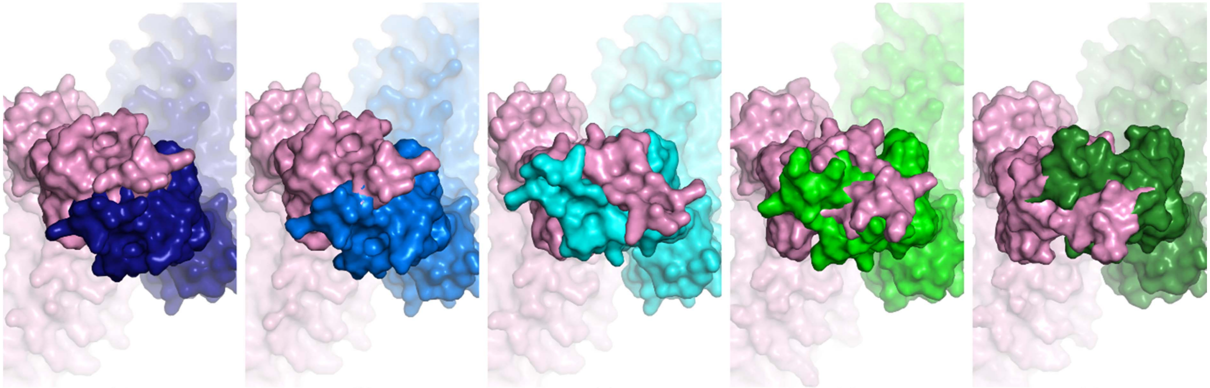

Figure S1 Clashes of two B repeats upon overlaying two $InlB_{392}$ molecules with the internalin domains in the full-length InlB dimer shown in Fig. 5. The view is from the top along the 2-fold axis of the dimer (rotated by 90° relative to the view in Fig. 5 along the horizontal axis). One chain of all $InlB_{392}$ structures was aligned with the grey monomer of full-length InlB as shown in Fig. 5. This chain is coloured as in Fig. 1, i.e. chains *A*, *B*, and *C* of $InlB_{392}$_wt are shown in dark blue, blue and cyan, respectively; crystal forms I and II of $InlB_{392}$_T332E are shown in green and dark green, respectively. A second chain of all $InlB_{392}$ structures was aligned with the pink monomer of full-length InlB as shown in Fig. 5. This second chain of $InlB_{392}$ is shown in pink in all cases. (*a*) $InlB_{392}$_wt, chain *A*. (*b*) $InlB_{392}$_wt, chain *B*. (*c*) $InlB_{392}$_wt, chain *C*. (*d*) $InlB_{392}$_T332E, crystal form I. (*e*) $InlB_{392}$_T332E, crystal form II.
